# Interlibrary loan and document delivery in North American health sciences libraries during the early months of the COVID-19 pandemic

**DOI:** 10.5195/jmla.2022.1452

**Published:** 2022-07-01

**Authors:** Jennifer K. Lloyd, Kristine M. Alpi, Margaret A. Hoogland, Priscilla L. Stephenson, Elizabeth Meyer

**Affiliations:** 1 jlloyd@lsuhsc.edu, LSU Health Sciences Center Libraries, New Orleans, LA.; 2 krisalpi@gmail.com, Associate Dean of Libraries & Information Sciences, Icahn School of Medicine at Mount Sinai, New York, NY.; 3 margaret.hoogland@utoledo.edu, University Libraries, University of Toledo, Toledo, OH.; 4 pstephenson9116@yahoo.com, James A. Haley Veterans' Hospital & Clinics, Tampa, FL.; 5 emeyer2018@gmail.com, Clinicient, Portland, OR.

**Keywords:** Access services, COVID-19, document delivery, health sciences libraries, hospital libraries, interlibrary loan, print collections

## Abstract

**Objective:**

The study purpose was to understand how early months of the COVID-19 pandemic altered interlibrary loan (ILL) and document delivery (DD) in North American health science libraries (HSLs), specifically the decision-making and workflow adjustments associated with accessing their own collections and obtaining content not available via ILL.

**Methods:**

Researchers distributed an online 26-question survey through 24 health science library email lists from January 6-February 7, 2021. Respondents reported their library's ILL and DD activities from March-August 2020, including ILL/DD usage and policies, collection access, decision-making, and workflow adjustments. In addition to calculating frequencies, cross-tabulation and statistical tests were performed to test a priori potential associations. Two researchers independently and thematically analyzed responses to the 2 open-ended questions and reached consensus on themes.

**Results:**

Hospital libraries represented 52% (n=226/431) of respondents, along with 42% academic (n=179) and 6% (n=26) multi-type or other special. Only 1% (n=5) closed completely with no remote services, but many, 45% (n=194), ceased ILL of print materials. More than half (n=246/423; 58%) agreed that ILL requests likely to be filled from print remained unfilled more than is typical. Open-ended questions yielded 5 themes on ILL/DD staffing, setup, and systems; 6 on impacts for libraries and library users.

**Conclusion:**

Lack of communication regarding collection availability and staffing resulted in delayed or unfilled requests. Hospital and academic libraries made similar decisions about continuing services but reported different experiences in areas such as purchasing digital content. Hybrid ILL/DD workflows may continue for managing these services.

## INTRODUCTION

Beginning in March 2020, many health sciences libraries (HSLs) in North America temporarily closed their physical locations for variable and unspecified lengths of time. Issues with receiving requests via interlibrary loan quickly became apparent. Requests were routed to closed libraries who had not marked themselves as inactive and also to libraries where staff working remotely did not have access to print collections.

The growing literature on library services during COVID-19 reflects several trends: rapid transition of services to a virtual environment, complications of restricted access to physical spaces and collections, navigating working from home, and development of solutions that will be retained after pandemic conditions cease. In the literature focused on Interlibrary loan / document delivery (ILL/DD) services, acquiring electronic resources to support virtual learning is another common theme [1, 2], as is the challenge of staggered staff schedules [1, 3]. Harnegie examined HSL staff experiences both working from home and in altered roles within hospitals [3], while Massey reported first-hand the stress of staff working at half capacity [4]. Koos et al. described the health and safety measures practiced by many HSLs during COVID-19, such as sanitation practices and quarantining of physical materials [1]. Prior disaster preparedness literature focused more on collection preservation than the library's role in the dissemination of information during a crisis [5]. The scale of the COVID-19 crisis may be unprecedented, but during prior public health crises, HSLs have planned for virtual learning and services and physical distancing [6]. Pivoting to the virtual environment in 2020 was challenging, especially with the short timeline, and extra responsibilities such as assisting teaching staff in transitioning courses online fell on library staff [7]. A more comprehensive review of literature relevant to this research, including articles on ILL/DD services outside of HSLs, appears in Appendix A.

Public dialogue about the impacts of COVID-19 on ILL and DD consisted primarily of posts on health sciences librarianship email discussion lists and social media, asking questions about service limitations and workarounds such as, “What are you doing about articles that you can't get through ILL at this time?” or “What are other academic health science resource libraries doing about filling ILL requests?” These posts provoked further questions about the increase in requests seen by HSLs that provided some level of service during COVID-19: “Was more health and science content needed during COVID-19? Was there a shift across the system that left fewer libraries to fill the same information needs? Or perhaps it was both?” To address these questions, a group of library personnel from multiple types of HSLs came together to investigate ILL and DD activities and associated collection access issues of HSLs during the pandemic [8]. The research was originally introduced as a “Print Collection Usage Study.” On April 3, 2020, DOCLINE released the new “Print Resources Available” filter to help address questions about which libraries still had access to their print collections [9], indicating that this was a major question for HSLs. However, subsequent dialogue among the team and questions from librarians with predominantly digital collections expanded the focus from ILL of print content to studying ILL/DD of materials regardless of format.

This survey is the first component of a multiple method program of interrelated research [8]. There are three method groups covering some complementary and some unique questions. The purpose of this survey was to understand ILL and DD activities in health-related libraries in North America during the early stage of the COVID-19 pandemic. The survey addressed the broad research question of understanding the decision-making and workflow adjustments associated with how libraries accessed their own collections and obtained content not available via ILL. The other two groups are analyzing secondary DOCLINE data and primary ILL and DD data from participating libraries over the same time period covered by our survey to be able to address questions about the type, age, subject, and digital availability of materials requested.

## METHODS

### Definitions

HSLs are libraries primarily serving health sciences programs, health professionals, or health facilities. ILL encompasses borrowing, obtaining digital or physical articles, chapters, books, or other materials from an external library for one's own users, or lending digital or physical articles, chapters, books, or other materials to other libraries. Libraries may borrow, lend, or do both. For the purpose of this study, DD is providing digital material (whether from digital collections or print) to a library's own users, unaffiliated requestors, or internal branches. The early stage of the COVID-19 pandemic is the time period from March through August 2020, as this was the most volatile time for libraries. This was also the time period in which the researchers hypothesized that the greatest difficulty in receiving requests occurred.

Organizations and systems of interest in this study include the National Library of Medicine (NLM) and its DOCLINE interlibrary loan system, which is reportedly used by at least 2,000 and up to 2,700 North American HSLs [10–11]. DOCLINE participation was considered as the baseline or denominator of available libraries engaged in health-related ILL as the majority of HSLs in the United States and Canada participate in DOCLINE. Unfortunately, the exact number of DOCLINE participants is not available. Some libraries, especially academics, also participate in OCLC Resource Sharing, an interlibrary loan network of over 10,000 libraries from more than 55 countries [12] or other cooperative ILL networks. DOCLINE and access to NLM collections were mentioned in the online forum questions that motivated this research. Therefore, we looked more closely at DOCLINE with this survey. Future research from the secondary data arm of our mixed-method research relies on DOCLINE data. The primary data arm of the research is examining ILL and DD data regardless of system [8].

### Study Design and Survey Development

This cross-sectional study consisted of a single online English-language survey to anonymously collect information about ILL and DD activities over the time period of interest. The research team developed a survey consisting of twenty-four multiple-choice and two open-response questions. Questions covered processes for filling ILL and DD requests from March-August 2020, using print and electronic methods to fill requests, copyright purchases, and operating functions of the library during the early months of the COVID-19 pandemic.

Five health sciences library staff familiar with ILL and working in public or private universities or hospitals completed the pilot survey in an average time of 15 minutes. Based on their comments and responses, the team revised the introduction, separated a few questions, and rewrote others for clarity. The final survey and the research protocol (STUDY00022454) were deemed exempt category #2 by the Oregon Health & Science University (OHSU) Institutional Review Board on January 4, 2021. The 26-question survey (Appendix B) was created in the OHSU-secure instance of Qualtrics online survey software.

### Study Population and Recruitment

Although the exact number of DOCLINE participants was not available, over 2,700 libraries in the US, Canada, and Mexico have reportedly participated in DOCLINE [11]. The research team recruited via general and regional English-language email discussion lists with subscribers from a wide range of HSLs from the United States, Canada, and Mexico. Based on the librarian response rate to other surveys of 10-20% [13], the potential number of DOCLINE libraries, and the bandwidth limitations for COVID survey participation, we hoped for responses from at least 100 academic and hospital libraries in North America. Survey instructions requested that the survey be completed only once per institution. Multi-campus institutions, which manage their own lending and borrowing, were asked to have each lending library participate individually in the survey. Because the email subscription lists represent individual library staff and not institutions, the number of DOCLINE libraries was used to approximate an institutional response rate.

The email with the survey link was distributed beginning January 6, 2021 to email discussion lists. Lists included those of the Association of Academic Health Sciences Libraries, MEDLIB-L, CANMEDLIB, six caucuses of the Medical Library Association (Hospital Libraries, Resource Sharing, Research, Leadership & Management, Consumer & Patient Health Information, Health Information & Corporate Librarians), the Association of College & Research Libraries Health Sciences Libraries Group, and a list specific for librarians of the Veterans Administration. Regionally the survey was shared with all MLA chapters through their email lists (PNC via HLIBPNW, Southern, South Central, NAHSL, NY-NJ, Midcontinental, Midwest, Hawaii-Pacific, Northern California and Nevada Medical Library Group, Philadelphia Regional Chapter, Upstate New York and Ontario Chapter, and Mid-Atlantic Chapter). The survey was open for one month, with reminders sent at two weeks and one week, resulting in three messages to each list by the survey end date of February 7, 2021. It was also distributed once to the DOCLINE-L email list on January 28, 2021. No participants were withdrawn from the survey responses received.

## ANALYSIS

Two researchers (EM, KA) reviewed all text responses to replace personal and institutional names or any other identifying text with generic terms prior to the responses being shared with other team members for analysis. Descriptive statistics were compiled for each question using Excel. Due to a priori assumptions that there would be differences between hospital and academic libraries, and that responses to several of the questions would be different for libraries with access to their print collections than those who did not have access, the researchers examined subgroups with cross-tabulations and Z-tests of proportions to assess whether differences were statistically significant.

Responses to the two open-ended questions covering decision-making strategies (Q25) and recommendations for additional research questions (Q26) were analyzed independently by two researchers (MH, KA). Respondents added comments about all aspects of the pandemic experience in response to both questions. Decision-making strategies were classified into discrete categories by two researchers (JL, PS), and agreement was reached by consensus to provide frequencies. Additional questions respondents thought should have been asked were extracted and aggregated (see Appendix C) to inform further research. For all other comments, two independent researchers (MH, KA) first used basic interpretive methods [14] of data reduction to code for potential themes. Many of the responses had to be parsed into codeable fragments, for example, this single response “No change in existing services or policies, other than we got busier and had to make extra efforts to find other libraries who were open and willing to provide ILL services. Had to change some settings in DOCLINE to route to libraries doing electronic.” The researchers then compared applied codes (Appendix D) and re-coded again using the constant comparative method [15]. The combined code listing was then applied to see if any of the responses offered insights that were not already covered by the previously created themes and to gather counts of coded fragments. The final analysis consisted of a list of agreed upon themes with counts and representative quotes. Participant quotes are followed by the respondent number in brackets, e.g. [R7].

## RESULTS

### Demographics

Ultimately 431 individuals completed the survey on behalf of their libraries. Of these, 414 reported participating in DOCLINE, thus the results could represent approximately 15.3% (414/2700) of DOCLINE-participating libraries, in addition to 17 libraries that did not participate in DOCLINE. All questions were optional, so the number of responses varied.

The majority of respondents (n=226/431; 52%) worked in hospital libraries, while 27% (n=116/431) worked in academic health science center libraries, and another 15% (n=63/431) in academic libraries supporting health sciences programs. The researchers reclassified 3 “Other” responses into the academic or hospital categories reported above. The remaining designated “Other” libraries 6% (n=26/431) either fit into multiple categories or were unique, e.g., government research, corporate, association, or other special libraries.

## LIBRARY OPERATIONS

**Figure 1:**
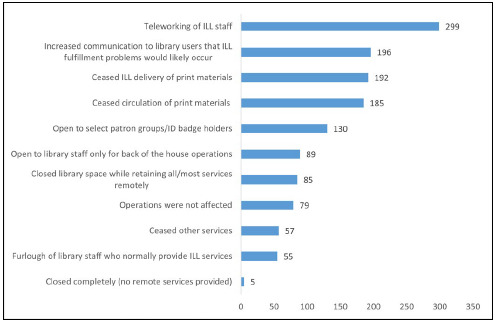
Percentage of Respondents (N=431) Reporting Various Impacts on Library Operations (1372 responses).

Figure 1 shows many changes to library operations during the early stages of the pandemic. Most respondents were affected in a variety of ways, but 18% (n=79/431) reported no effect on operations. ILL staff teleworking was the most common response at 69% (n=299/431), while 13% (n=55/431) experienced a furlough of library staff who normally provide ILL services. Of responding libraries that furloughed staff, 62% (n=34/55) were hospital libraries, 31% (n=17/55) were academic or academic health sciences libraries, and 7% (n=4/55) were other types of health science libraries.

Lack of access to print materials was linked to the cessation of ILL/DD for print materials in 45% (n=192/431) of libraries, and 43% (n=185/431) reported that circulation of print items ceased. Library facilities were open only to select patron groups or badge holders for 30% (n=130/431), and open only to library staff for 21% (n=89/431). Another 20% (n=85/431) of the facilities were closed, with most or all of their services continuing remotely, while 1% (n=5/431) were completely closed with no remote services.

Most libraries (n=365/429; 85%) reported that they filled ILLs with electronic subscriptions during the entire time. Another 9% (n=39/429) did part of the time, and 5% (n=25/429) did not at any point in the study period. The majority of respondents (n=414/428; 97%) used DOCLINE for ILLs, 49% (n=208/428) used OCLC, 18% (n=75/428) used regional or state ILL networks, 6% (n=26/428) used RapidILL, and 4% (n=17) reported using other methods. Libraries used DOCLINE (n=394/428; 91%), OCLC (n=173/428; 40%), and direct email, or other non-DOCLINE or OCLC request from the requesting library (n=117/428; 27%) to deliver items. Other methods accounted for 15% of responses.

**Figure 2:**
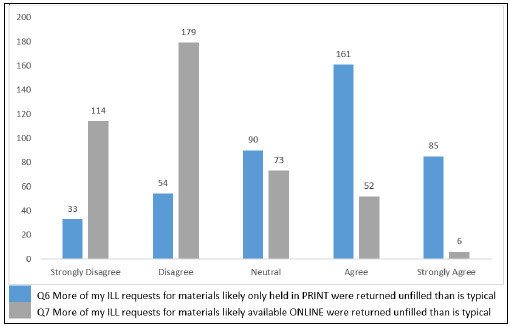
Perception of Unfilled Requests: Print vs Electronic.

Figure 2 shows respondents' perceptions about unfilled requests on a 5-point scale from strongly disagree to strongly agree. Regarding whether more print ILL requests were returned unfilled than was typical, 58% agreed or strongly agreed (n=246/423), 21% disagreed or strongly disagreed (n=87/423), and 21% were neutral (n=90/423). When asked if more online ILL requests were returned unfilled than was typical, only 13% of the respondents agreed or strongly agreed (n=58/424), while 69% disagreed or strongly disagreed (n=293/424), and another 17% were neutral (n=73/424).

While a majority of respondents (n=282/423; 67%) did not purchase digital versions of requested items not available through ILL, some (n=99/423; 23%) purchased items directly from publishers or purchased items through an existing arrangement with a third-party provider (n=62/423; 15%). This question did not include existing subscriptions.

Many libraries reported access to their physical collections part of the time (n=194/429; 45%) and filled requests part of the time (n=174/430; 40%) from their physical collections. Some libraries had access to their physical collections the entire time (n=124/429; 29%) and filled ILL requests the entire time (n=104/430; 24%). Of the 108 (25%) libraries who did not fill ILL requests, 65 (15%) of these libraries had no access to their physical collections. Forty-six respondents (11%) selected “Not applicable,” because their libraries had no physical collections and therefore could not fill ILL print requests. For those who responded “Other” to these questions, the researchers re-coded the responses into the appropriate categories. Hospital libraries (15%, n=33/227) were more likely to rely solely on virtual collections (z=3.0, p=.0027) than all other types of libraries combined (6%, n=13/202).

**Figure 3:**
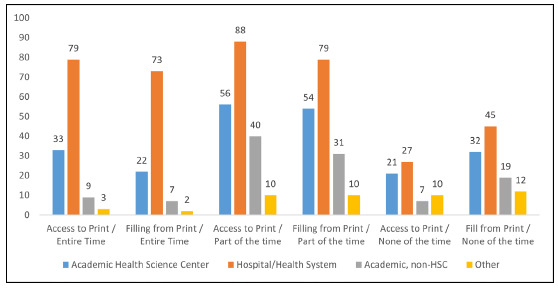
Print Collection Access (N=429) and Print ILL Participation (N=430) by Type of Library. *

*Not shown are the excluded 46 not-applicable responses for print collection access and 44 not-applicable responses for print ILL participation.

The majority of libraries (n=289/389; 74%) provided ILLs from their physical collections and scanned their own physical documents for digital document delivery. For DD, 32% used their physical collections to fill DD requests all of the time (n=129/431; 32%), 45% (n=183/431) did some of the time, and 23% (n=95/431) did not use their physical collections at all. Most libraries used their online collections to fill DD requests all of the time (n=373/431; 89%) and an additional 7% some of the time (n=32/431). Only a few libraries (n=10/431; 2%) did not fill DD requests from their online collections. Over a third (n=156/431; 36%) did not alter their ILL and DD workflows. Some libraries (n=111/431; 26%) offered ILL services with an altered schedule. Another 19% (n=81/431) responded either “not applicable” or “not scanning ILL/DD,” and 14% (n=59/431) selected “other.”

In terms of managing print materials used for ILL with workflow and available staff changes, half (n=206/411; 50%) reported no change in reshelving print materials. Of the 50 who delayed reshelving, 12% (n=50/411) created a system for locating materials not housed in their regular locations, while 10% (n=40/411) did not. For those 65 respondents who did not reshelve at all during this time period, 12% (n=50/411) did not create a system, and 4% (n=15/411) did. While the survey did not ask about quarantining due to the many changes in guidance, in the open-ended responses several mentioned quarantining materials for various lengths of time.

Some libraries (n=120/431; 27%) loaned physical items to their patrons the entire time. Fewer than half of the libraries (n=173/431; 40%) loaned physical items to their own patrons part of the time. Additionally, some libraries (n=99/431; 23%) did not loan physical items, and 8% (n=38/431) lacked physical collections. Some (n=121/407; 29%) offered in-library pickup of physical items to patrons, 25% (n=101/407) used campus mail to deliver physical items to patrons, and 26% (n=105/407) did not loan physical items. Almost half, 45% (n=195/431), accepted returned physical items the entire time, 24% (n=102/431) accepted returns only part of the time, and 13% (n=57/431) did not accept returns. For the 11% selecting “not applicable” (n=50/431), the team concluded these libraries did not lend physical materials.

### Library Collection and Service Evaluation

**Figure 4:**
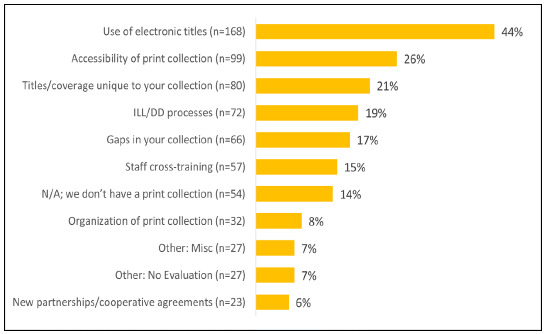
Percentage of Respondents (N=383) Evaluating Print Collection (705 responses).

Figure 4 shows responses from a number of provided options regarding collection evaluation and usage of print collections for ILL/DD. The most common response (n=168/383; 44%) was that the pandemic caused them to evaluate the use of electronic titles. Another 99 (26%) were evaluating the accessibility of their print collections. Eighty (21%) were evaluating unique titles and subject coverage in their collections. Seventy-two (19%) were evaluating their ILL and DD processes. Another 66 (17%) were evaluating gaps in their collections. Answers about staff cross-training, ‘not applicable' and ‘other,' were each approximately 14%. Only 32 (8%) had evaluated the organization of their print collections, and fewer (23; 6%) had pursued new partnerships or cooperative agreements with other libraries. Of the other responses, 27 (7%) said that no evaluation had been conducted. Despite the large number of responses, 48 participants did not answer the question.

Several questions addressed policies and practices on copyright and fees. Regarding interpretation of copyright law or practices using CONTU Guidelines (guidelines for copying from periodicals which apply to materials less than 5 years old; also referred to as “rule of five”) during the pandemic, 79% (n=339/428) had not changed their interpretation, while 13% (n=57/428) had, and another 6% (n=26/428) were considering a re-evaluation of how they interpreted complying with copyright such as moving from following the CONTU guidelines to a more holistic review of requests. Two respondents from outside the United States indicated they operated under different national copyright laws.

Most (87%; n=372/429) had not changed their policies for not charging for DD, and 9% (n=39/429) who did charge also had not changed their policies. Only 4% (n=17/429) changed their DD fees to provide free or lower cost documents for some or all patrons. Most hospital libraries did not change their no-charge policies for DD, with 91% (n=206/226) reporting no change, 7% (n=16/226) did not change their policy of charging, and 2% (n=4/226) changed their DD fees to provide free or lower cost items.

When asked about the financial impact on ILL, nearly a quarter of respondents did not have access to this information (n=105/426; 25% for borrowing and n=119/423; 28% for lending). Regarding lending revenue, 10% (n=44/423) reported a decrease and 4% (n=17/423) an increase. A slight majority reported no change for borrowing (n=238/426; 56%) or lending (n=243/423; 57%). Only 51 said borrowing expenditures increased, and 32 said borrowing expenditures decreased. Hospital libraries generally reported no change for borrowing (n=141/225; 63%) while 8% (n=18/225) noted a decrease, and 9% (n=21/225) experienced an increase. A related question (Q8) about financial impact asked about purchasing access to materials, where nearly 67% (n=282/423) of respondents said that they did not purchase digital access to any materials. Most hospital libraries (n=178/223; 80%) did not purchase digital access to any requested materials they could not obtain. Compared with 50% (n=87/174) of academic libraries, there is a statistically significant difference (z=6.3, p<0.0001) in purchasing digital access between the two types of libraries.

For the free text question about ILL staff involvement in service decisions, of the 288 codable responses, 258 could be classified. The others gave examples of things that had changed without directly addressing the decision-making strategy. “Group decision” (either through teams or meetings) was the most common response (n=89/258; 34%), with a substantial number of solo librarians (n=51/258; 20%) making their own decisions. Involvement of ILL staff in decision making was common (n=36/258; 14%), but so was having decisions being directed (n=42/258; 16%) either through top-down decisions by others, or no involvement by ILL staff. Another 40 reported not making service changes. Although the survey did not ask about staff size, 64 respondents mentioned that they were solo librarians or solo ILL staff, with some mentioning small staff size as being a factor in decision making. While discussing changes, respondents mentioned the challenges of running ILL while they or their staff were furloughed.

The final open-ended question, which invited additional comments and other questions that respondents felt the survey should have asked, elicited 162 responses. Beyond responses like “no” and “thank you,” 118 in-depth comments were analyzed. Staff size and associated staffing and workflow issues, as well as fill-rate questions, predominated the questions that respondents thought should have been asked. These are categorized into 15 areas presented in Appendix C. The thematic analysis of the general comments from the 2 open-ended questions were clustered into 2 high-level categories shown with associated themes and representative quotes in Table 1. The first, “ILL/DD Staffing, Set Up, and Systems,” had 5 themes, the most prominent of these was “ILL staffing changes and cross training.” Respondents also commented on how they worked with the DOCLINE system more than with other ILL systems. The largest category was “Impact on Libraries and Library Users,” which encompassed 6 themes. The most frequently categorized comments included: change in lending demand, participation, fill rate, turnaround times. Researchers frequently assigned these categories in addition to themes of either negative impact of lack of access via ILL or access limitations to one's own collection.

**Table 1:** Qualitative analysis of open-ended questions (Q25, N=258 and Q26, N=118) represented by eleven themes with representative quotes. Q25. How were ILL staff involved in making decisions about any changes that happened with services? Q26. Is there anything else you would like us to know or think we should have asked?

Theme	Times coded* - Q25	Times coded* - Q26	Representative quote(s) [Question and respondent number]
ILL/DD STAFFING, SET UP AND SYSTEMS			
Communication among libraries	4	4	…based on some teleconferences, and listserv discussions on what the majority of what the other libraries were doing. [Q25; R159]
DOCLINE customization, performance, and status changes	6	9	It was very tedious to have to keep running things through Docline again and again, until they added some tools to make it easier to tell who has access to their print collections. [Q25; R51]
ILL staffing change and cross training	18	7	…ILL staff handled all remote training for one staff member from the access services department who was working onsite. That staff member handles all the scanning from our print collection for borrowing and lending. [Q25; R67]
Sharing system customization and performance, nonDOCLINE	3	5	I appreciated that OCLC created new custom groups (ACOV, BCOV and CCOV) for libraries that were still able to lend print, ebooks, or items from physical collections. [Q25; R280]
Setting up/changing ILL technology for remote work	5	4	ILL staff implemented a paperless process a month before Pandemic hit, so were able to positively impact the ability of our staff to provide IIL service while working at home for ILL delivery and electronic collection access. [Q25; R12]
IMPACT ON LIBRARIES AND LIBRARY USERS			
Access limitations to own library's physical collection	3	11	The experience of not having full access to the print collection from March to August reinforced that certain content remains only available via print. [Q26; R148]
Change in lending, demand, participation, demand, fill rate, turnaround times	6	18	…Except for decreased ILL lending, nothing substantially changed over that time period [Q25; R203]
Financial implications**	5	5	…Additionally, our staff lobbied for and were allowed to lend freely to other libraries that also lent freely. [Q25; R35]
Negative impact of lack of access via ILL	1	13	…More of my requests were canceled, especially those to NLM [National Library of Medicine], during the pandemic. This has affected access to information for the hospital staff. [Q26; R9]
Perceived value of library services	1	7	…When I returned from furlough, I was shocked to learn how many libraries [had] completely closed their print collection. It left those of us at smaller systems and with limited budgets in quite a bind.…I've rarely had to tell a customer I was unable to get what they needed but had (and still have) to do it often [during this period]. It bothers me very much, yet it has helped customers realize how vital libraries and interlibrary loan processes are to everyone. My biggest concern is perception - if libraries close during a pandemic when people need us the most, I feel we've shot ourselves in the foot. [Q26; R380]
User needs, attitudes, and responses	1	7	…we garnered a whole new population of library users because [UNIT NAME REDACTED] relocated their home base to inside the Medical Library when their offices on the floors were displaced to become a closed COVID-19 unit [Q26; R110].

* Times coded may add up to more than the total number of responses, as most responses include multiple codable fragments.

** Covered a broad range of topics, so no single quote is truly an exemplar: comments about charging users for services, advocating for free lending partners, canceling or adding journal subscriptions, shipping costs, and budgets impacted by closures.

In some cases, the researchers focused the themes narrowly to provide actionable insight, e.g., sorting the system customization and performance issues into DOCLINE and non-DOCLINE. On the other hand, the theme “Financial Implications” covers a broad range of financial situations assigned to comments about charging users for services, advocating for free lending partners, canceling or adding journal subscriptions, shipping costs, and budgets impacted by closures. In this case, no single quote provided a representative example. There were many other topics mentioned, such as “physical material handling” and “digital resource purchasing” that were not coded, since these questions were asked in the survey.

## DISCUSSION

The survey findings support existing trends that health sciences libraries rely on digital collections of current materials [16], with 46/429 (11%) of respondents reporting only providing digital collections and 33 of these being hospital libraries. Almost all libraries (94%) provided ILL and DD from their online collections at least part of the time. As 69% (n=299/431) of ILL staff were teleworking during at least part of this period, it is not surprising that in the open-ended comments, libraries discussed the challenges in setting up existing ILL system software to work from home. Improvements to these systems to facilitate working off-site and eliminating licensed resource clauses requiring printing and re-scanning are important to working sustainably.

Although this survey was not designed to highlight differences between hospital libraries and academic health sciences libraries, many comments invited a closer look at how the pandemic experience varied by library type. Solo librarians and small hospital libraries transitioned more smoothly to ILL/DD as remote workers as their ILL operations primarily relied on digital materials, and they did not have to coordinate across multiple staff. Academic libraries, by contrast, transitioned more slowly and in many cases with greater difficulty. Changes from in-person to remote staffing and cross-training of staff members are two examples of transition difficulties and delays in ILL/DD provided by academic HSLs. By contrast, most smaller libraries and solo librarians simply shifted the time spent on these services. In summarizing library work in a virtual environment during the pandemic, Koos pointed to collection management of physical items and interlibrary loan digitization as two of the library operations requiring on-site staffing [1], both areas that disappear with entirely digital collections.

Some libraries issued top-down directives regarding changes to existing ILL/DD services with little or no input from ILL staff. It is unclear if these directives were issued by library administrators or non-library administrators. The impact of decisions made by others seems to have particularly affected staff morale and the ability of staff to continue to do what was needed to meet the needs of library users. Massey mentions that for staff working a staggered schedule in a general academic library, leaving piles of work behind made staff feel like they were not doing enough [4]. In the present study there were responses from furloughed solo staff who had to leave their work with little notice for much longer periods. Both the volume and depth of responses were surprising, particularly for the multiple selection questions and open-ended responses. The survey never intended to describe the morale and emotional impact of working through the pandemic, and therefore the researchers found it challenging to distinguish the influence of the COVID-19 pandemic on services from its influence on staff attempting to provide the best service possible amidst these challenging circumstances.

Our survey likely underestimates the impact of furloughs on ILL services provided by solo librarians as furloughed librarians may not have seen the invitation to participate. The survey was only available in English, and therefore our results may not represent North American libraries in Canada and Mexico where French and Spanish languages predominate. We welcome translation and reproduction of the survey. As the varying number of respondents per question shows, respondents skipped questions that they felt did not apply. As part of the open-ended responses, a few participants remarked about questions that were confusing or hard to answer; two of these answers related to definitions used in the study, and two related to answer options. Rather than separating print journals and print books, the survey asked about print collections. As some respondents mentioned having print books but not print journals, responses might not have meant to include both journals and books. The study defined ILL as referring to loans and copies for other libraries and DD as supplying documents for one's own clients. These definitions did not always match how those terms were used by a few respondents, as noted by this respondent: “The demarcation you're making between ILL and DD is vague and confusing, so I found portions of the survey to feel either not applicable or like there wasn't an option available to select. In our hospital library, ILL is typically only print materials, while DD covers everything else—this has nothing to do with whether the request is coming from an internal or external client.” [Q26; R246] For response options, the library access question did not provide sufficient choices to describe the variety of arrangements used to come on site to meet users' needs. The question about expenditures did not reflect the experience of libraries that only request items from free reciprocal sources. Additionally, due to Electronic Fund Transfer System (EFTS) service migrations in the summer of 2020, participating libraries may not have had the reports to answer this question. In the interpretation of responses, for those reporting increased expenditures, it was not possible to distinguish whether this was a function of increased volume of requests with prices holding steady, increased prices for obtaining materials, or a combination of both volume and price increases.

The research team intended the survey to be relevant to all sizes and structures of health science libraries. A number of respondents reported that the survey seemed to assume staff were dedicated to ILL or DD, and therefore the survey was intended for larger or academic libraries rather than those directed by solo librarians or small teams. The survey did not ask about the size of the library or organization or the number of library full time employees though many respondents commented that they were solo-staffed or small libraries in the open-ended responses. It was suggested that future research ask about library and staff size; see the categorized list of suggested questions for further research provided in Appendix C.

The research team expected to learn from the respondents about the intersection of physical and digital collections with on-site and virtual ILL and DD services. Perceptions of limited access to physical collections was a primary driver for this research. This report brings together responses to direct questions about access to library collections and open-ended comments about challenges regarding ILL borrowing requests likely to be filled from print and increases in lending demand for libraries that were able to fill from their collections during this time. It also painted a picture regarding the impact of the NLM and other large libraries not filling loan or copy/scan requests from their print collections. Few libraries pursued new cooperative agreements to try to increase access to resources, but we do not know why–it could be they viewed existing arrangements as adequate, that they lacked funds or capacity to pursue anything, or there was no one to pursue, because all of libraries were impacted due to the global nature of the pandemic.

This makes the assessment of what content is needed but not yet digitally available more critical for libraries and content producers. Even if all content were digitally available, there remain questions of financial capacity and impact. As discussed above, financial impacts were not fully assessed in this survey, and more detailed information would be needed to truly answer questions about the financial impacts of the pandemic on library collections and service expenditures.

Finally, given the challenges of the pandemic, it was not surprising that so many of the comments to the survey's open-ended questions focused on negative impacts or overcoming difficulties outside of ILL/DD services. The survey became an outlet for grief for some respondents. To remain focused on the original research questions, the team decided not to analyze these comments, while still recognizing the pain of fellow librarians. The data is available should other researchers wish to study these comments. The following quote is one of the positive comments offered by a respondent: “… Our objective was to make library services seamless, no matter where we were physically located. I know we succeeded. The medical library was referred to as one of the gold stars of the Hospital during the pandemic.” [Q25; R7]

## CONCLUSION

The survey team is one part of a multi-component research strategy regarding COVID-19 and ILL/DD activities. The survey team shared the questions and concerns with the other research teams for their consideration. The studies planned by the other teams, along with a wealth of other pandemic-related surveys and publications in progress by other researchers, should shed further light on questions raised by these responses.

The most powerful finding is that resource sharing continued in 94% (n=404/429) of responding libraries and most agreed that their needs for materials likely to be provided digitally were met. While there were frustrations, requests were filled more often than not. This speaks to the dedicated librarians and library staff who persisted in managing ILL and DD services despite unprecedented challenges.

## Data Availability

Data associated with this article are available in the Oregon Health & Science University Library Digital Collections institutional repository at https://doi.org/10.6083/000000663. Per the consent under which the data was collected, there are separate, unlinked files for the closed-ended and open-ended question responses to preserve respondent anonymity. Researchers interested in accessing the linked data should contact Kristine Alpi.

## SUPPLEMENTAL FILES

**Appendix A:** Literature Review

**Appendix B:** Original Survey

**Appendix C:** List of Further Research Questions Raised by Respondents

**Appendix D:** List of All Codes Considered and Applied to Data from Q25 and Q26

## References

[1] Koos J, Scheinfeld L, Larson C. Pandemic-proofing your library: disaster response and lessons learned from COVID-19. Med Ref Serv Q. 2020 Feb;40(1):67–78. DOI: 10.1080/02763869.2021.1873624. 33625324

[2] Howes L, Ferrell L, Pettys G, Roloff A. Adapting to remote library services during COVID-19. Med Ref Serv Q. 2021 Feb;40(1):35–47. DOI: 10.1080/02763869.2021.1873616. 33625328

[3] Harnegie MP. COVID snapshot: How medical libraries and staff adapt to deliver services during a pandemic. J Hosp Librariansh. 2021; 21(2):173–183. DOI: 10.1080/15323269.2021.1904184.

[4] Massey ME. Lessons learned in leaving the library and coming back again. Pennsylvania Libraries: Research & Practice. 2020 Fall;8(2):100–102. DOI: 10.5195/palrap.2020.239.

[5] Clifton VL, Flathers KM, Brigham TJ. COVID-19: Background and health sciences library response during the first months of the pandemic. Med Ref Serv Q. 2021 Feb;40(1):1–10. DOI: 10.1080/02763869.2021.1873611. 33625334

[6] Norton MJ, Wilson DT, Yowell SS. Partnering to promote service continuity in the event of an emergency: a successful collaboration between two interlibrary loan departments. J Med Libr Assoc. 2009 Apr;97(2):131–134. DOI: 10.3163/1536-5050.97.2.010. 19404504 PMC2670208

[7] Tranfield MW, Worsham D, Mody N. When you only have a week: rapid-response, grassroots public services for access, wellness, and student success. C&RL News. 2020 Jul/Aug;81(7):326–329, 336. DOI: https://crln.acrl.org/index.php/crlnews/article/view/24533.

[8] Creazzo J, Bakker C, Jo P, Koos J, Alpi KM. Report from the Field: Researching interlibrary loan/document delivery usage by health sciences libraries during the COVID-19 pandemic. Journal of Interlibrary Loan, Document Delivery & Electronic Reserve. 2020;29(3-5):171–179. DOI: 10.1080/1072303X.2021.1936739.

[9] Tamase M. DOCLINE update: new “print resources available” filter now available! [Internet]. 2020 April 3 [cited 2021 September 29]. https://news.nnlm.gov/psrnewsbits/docline-print-resources-available-filter/.

[10] Theisen L. DOCLINE: connecting medical libraries for 35 years [Internet]. 2020 December 16 [cited 2021 October 12]. https://nlmdirector.nlm.nih.gov/2020/12/16/docline-connecting-medical-libraries-for-35-years/.

[11] National Library of Medicine. Programs and Services Fiscal Year 2012. [Internet]. 2012 [cited 2021 October 11]. https://www.nlm.nih.gov/ocpl/anreports/fy2012.pdf.

[12] OCLC resource sharing facts and statistics [Internet]. 2021 [cited 2021 October 11]. https://www.oclc.org/en/worldshare-ill/statistics.html.

[13] Lessick S, Perryman C, Billman BL, Alpi KM, De Groote SL, Babin TD Jr. Research engagement of health sciences librarians: a survey of research-related activities and attitudes. J Med Libr Assoc. 2016 Apr;104(2):166–73. doi: 10.3163/1536-5050.104.2.015. 27076808 PMC4816469

[14] Merriam S. Qualitative research in practice. San Francisco, CA: Jossey-Bass; 2002.

[15] Strauss A, Corbin J. Basics of qualitative research: grounded theory procedures and techniques. Newbury Park, CA: Sage; 1990.

[16] Burrows, S. A review of electronic journal acquisition, management, and use in health sciences libraries. J Med Libr Assoc. 2006 Jan;94(1):67–74. PMID: 16404472; PMCID: 16404472. 16404472 PMC1324774

