# Supplementary material for: Interlibrary loan and document delivery in North American health sciences libraries during the early months of the COVID-19 pandemic
Source: J Med Libr Assoc. 2022 Jul 1;110(3):348–57. doi: 10.5195/jmla.2022.1452 (PMC9782377; doi:10.5195/jmla.2022.1452)
Supplement: Supplementary file 1 — Appendix A: Literature Review [file jmla-110-3-348-s01.pdf]

Appendix A: Literature Review

Brown HL. Pay-per-view in interlibrary loan: a case study. J Med Libr Assoc. 2012 Apr;100(2):98–103. DOI: https://doi.org/10.3163/1536-5050.100.2.007.

Chan EK, Mune C, Wang Y, Kendall SL. Three years of unmediated document delivery: an analysis and consideration of collection development priorities. Med Ref Serv Q. 2016 Jan;35(1):42–51. DOI: https://doi.org/10.1080/02763869.2016.1117288.

Clifton VL, Flathers KM, Brigham TJ. COVID-19: Background and health sciences library response during the first months of the pandemic. Med Ref Serv Q. 2021 Feb;40(1):1–10. DOI: https://doi.org/10.1080/02763869.2021.1873611.

Creazzo J, Bakker C, Jo P, Koos J, Alpi KM. Report from the Field: Researching interlibrary loan/document delivery usage by health sciences libraries during the COVID-19 pandemic. Journal of Interlibrary Loan, Document Delivery & Electronic Reserve. 2020;29(3-5):171-179. DOI: 10.1080/1072303X.2021.1936739.

Donahue AE, Featherstone RM. New roles for hospital librarians: a benchmarking survey of disaster management activities. J Med Libr Assoc. 2013 Oct;101(4):315–318. DOI: https://dx.doi.org/10.3163/1536-5050.101.4.014.

Featherstone RM, Boldt RG, Torabi N, Konrad SL. Provision of pandemic disease information by health sciences librarians: a multisite comparative case series. J Med Libr Assoc. 2012 Apr;100(2):104–112. DOI: 10.3163/1536-5050.100.2.008.

Fought RL. Breaking inertia: increasing access to journals during a period of declining budgets: a case study. J Med Libr Assoc. 2014 Jul;102(3):192–196. DOI: https://doi.org/10.3163/1536-5050.102.3.009.

Frederick JK, Wolff-Eisenberg C. Academic library strategy and budgeting during the COVID-19 pandemic. ITHAKA S+R [Internet]. ITHAKA S+R; 9 Dec 2020 [cited 28 Apr 2021]. DOI: https://doi.org/10.18665/sr.314507.

Gotschall T, Gillum S, Herring P, Lambert C, Collins R, Dexter N. When one library door closes, another virtual one opens: a team response to the remote library. Med Ref Serv Q. 2021 Feb;40(1):11–22. DOI: https://doi.org/10.1080/02763869.2021.1873612.

Harnegie MP. Evaluation of library usage and attitudes of residents and fellows: results of 2017-2019 surveys. J Hosp Librariansh. 2021; 21(2): 141–157. DOI: https://doi.org/10.1080/15323269.2021.1899781.

Harnegie MP. COVID snapshot: How medical libraries and staff adapt to deliver services during a pandemic. J Hosp Librariansh. 2021; 21(2):173–183. DOI: https://doi.org/10.1080/15323269.2021.1904184.

Haugh D. Communicating with medical library users during COVID-19. J Med Libr Assoc. 2021 Jan;109(1):107–111. DOI: https://doi.org/10.5195/jmla.2021.1003.

Hendler GY, Gudenas J. Developing collections with get it now: a pilot project for a hybrid collection. Med Ref Serv Q. 2016 Sep;35(4):363–371. DOI: https://doi.org/10.1080/02763869.2016.1220751.

Howes L, Ferrell L, Pettys G, Roloff A. Adapting to remote library services during COVID-19. Med Ref Serv Q. 2021 Feb;40(1):35–47. DOI: https://doi.org/10.1080/02763869.2021.1873616.

Jarvis C, Marcotte Gregory J, Mortensen-Hayes A, McFarland M. Borrowing trouble: the impact of a systematic review service on interlibrary loan borrowing in an academic health sciences library. J Med Libr Assoc. 2021 Jan;109(1):84–89. DOI: https://dx.doi.org/10.5195/jmla.2021.1005.

Kehnemuyi K, Larsen SC. Shadow ILL services: how scholarly pirate websites and hacking affect ILL. J Interlibrary Loan Document Delivery & Electronic Reserve. 2020 Apr;28(5):139–149. DOI: https://doi.org/10.1080/1072303X.2020.1749750.

Koos J, Scheinfeld L, Larson C. Pandemic-proofing your library: disaster response and lessons learned from COVID-19. Med Ref Serv Q. 2020 Feb;40(1):67–78. DOI: https://doi.org/10.1080/02763869.2021.1873624.

Kraft M. When can we get articles from NLM print collection?! In: MEDLIB-Listserv. [Internet]. University of Vermont. 2021 Jul 14. [cited 2021 Dec 15].

Larsen SC, Gibson DS. Checking in with your document delivery user base: creating, implementing, and learning from client satisfaction surveys. Med Ref Serv Q. 2020 Apr;39(2):153–164. DOI: https://doi.org/10.1080/02763869.2020.1741307.

Massey, ME. Lessons learned in leaving the library and coming back again. Pennsylvania Libraries: Research & Practice. 2020 Fall;8(2):100–102. DOI: https://doi.org/10.5195/palrap.2020.239.

McCaslin D. What are the expectations of interlibrary loan and electronic reserves during an economic crisis? J Interlibrary Loan, Document Delivery & Electronic Reserve. 2010 Sept;20(4):227–231. DOI: https://doi.org/10.1080/1072303X.2010.507722.

McGuire L. Planning for a pandemic influenza outbreak: roles for librarian liaisons in emergency delivery of education programs. Med Ref Serv Q. 2007;26(4):1–13. DOI: https://doi.org/10.1300/J115v26n04_01.

Medical Libraries Association. Disaster information specialization. MLANET [Internet]. Chicago, IL: The Association [cited 21 Apr 2021]. https://www.mlanet.org/p/cm/ld/fid=338.

Mi M, Zhang Y, Wu L, Wu W. Four health sciences librarians' experiences: how they responded to the COVID-19 pandemic crisis. C&RL News. 2020 Jul/Aug;81(7): 330–334. DOI: https://doi.org/10.5860/crln.81.7.330.

Nash JL, McElfresh KR. A journal cancellation survey and resulting impact on interlibrary loan. J Med Libr Assoc. 2016 Oct;104(4): 296–301. DOI: https://dx.doi.org/10.3163/1536-5050.104.4.008.

National Library of Medicine. About the Disaster Information Management Resource Center (DIMRC) [Internet]. Disaster Information Management Resource Center [rev. 5 Dec 2019, cited 21 Apr 2021]. https://disasterinfo.nlm.nih.gov/about-dimrc.

Norton MJ, Wilson DT, Yowell SS. Partnering to promote service continuity in the event of an emergency: a successful collaboration between two interlibrary loan departments. J Med Libr Assoc. 2009 Apr;97(2):131–134. DOI: https://doi.org/10.3163/1536-5050.97.2.010.

Pierard C, Shoup J, Clement SK, Emmons M, Neely TY, Wilkinson FC. Building back better libraries: improving planning amidst disasters. Advances in library administration and organization, Hines SS, Crowe KM, eds, vol. 36. Emerald Group Publishing Limited; 2016. 307–333. DOI: https://doi.org/10.1108/S0732-067120160000036014.

Ragon B, Whipple EC, Rethlefsen ML. Impact of Covid-19 on academic health sciences library programs and services. Medical Library Association Annual Meeting, online. 5/24/2021.

RapidILL thank you COVID19 pod. https://page.exlibrisgroup.com/hubfs/EMEA/RapidILL/RapidILL%20Thank%20You%20COVID19%20Pod.pdf.

Tranfield MW, Worsham D, Mody N. When you only have a week: rapid-response, grassroots public services for access, wellness, and student success. C&RL News. 2020 Jul/Aug;81(7):326–329, 336. DOI: https://crln.acrl.org/index.php/crlnews/article/view/24533.

Weeks A, Houk KM, Nugent RL, Corn M, Lackey M. UNLV health sciences library's initial response to the COVID-19 pandemic: how a versatile environment, online technologies, and liaison expertise prepared library faculty in supporting its user community. Med Ref Serv Q. 2020 Oct;39(4):344–358. DOI: https://doi.org/10.1080/02763869.2020.1826197.
