# Supplementary material for: Interlibrary loan and document delivery in North American health sciences libraries during the early months of the COVID-19 pandemic
Source: J Med Libr Assoc. 2022 Jul 1;110(3):348–57. doi: 10.5195/jmla.2022.1452 (PMC9782377; doi:10.5195/jmla.2022.1452)
Supplement: Supplementary file 2 — Appendix B: Original Survey [file jmla-110-3-348-s02.pdf]

Appendix B: Original Survey

COVID ILL/DD Survey
The purpose of this survey is to understand Interlibrary Loan (ILL) and Document Delivery (DD) activities in health-related libraries during the early stage of the COVID-19 pandemic in North America. For the purposes of this survey, ILL is defined as lending/borrowing to/from external libraries and DD as providing material to one's own users, unaffiliated requestors, or internal branches. We are asking about the time period from March 2020 through August 2020.

All health-related libraries (those who serve health sciences programs, health professionals and/or health care facilities), are invited to participate. Please complete the survey once per institution. If your institution has more than one location that does their own lending, each lending location should complete the survey

1. What is your type of library? Select the best choice.
___Academic Health Science Center/Academic Medical Center
___Academic (supporting health programs, but not at a health sciences or medical center)
___Association, Health or Medical-related
___Hospital/Health System (including government systems, e.g., Veterans Administration)
___Special (e.g. law, corporate, etc.)
___Other ___________

2. How were your library's operations affected by COVID-19? Please select all that applied at any point from March to August 2020.
___Operations were not affected
___Ceased circulation of print materials
___Ceased ILL delivery of print materials
___Increased communication to library users that ILL fulfillment problems would likely occur due to the pandemic closures of other libraries
___Ceased other services, please specify: ____
___Open to select patron groups/ID badge holders
___Open to library staff only for back of the house operations
___Teleworking of ILL staff
___Closed library space while retaining all/most services remotely
___Furlough of library staff who normally provide ILL services
___Closed completely (no remote services provided)

3. Were you providing ILL from your electronic subscriptions at any time from March to August 2020? Select one choice.
___ Entire time
___ Part of the time
___ None of the time
___ We do not have any electronic subscriptions or our subscriptions do not allow ILL.

4. In which of these ILL systems do you normally participate? Select all that apply.
___DOCLINE
___OCLC
___RapidILL
___Regional or state system
___Other __________

5. How did you receive ILL lending requests from March to August 2020? Select all that apply.
___Direct email or other non-system request from borrowing library
___DOCLINE
___OCLC
___RapidILL
___Regional or state system
___ Other ___________
___ Not applicable, we did not receive ILL requests during that time

6. More of my ILL requests for materials likely only held in print, were returned unfilled than is typical
___Strongly disagree
___Disagree
___Neutral
___Agree
___Strongly agree

7. More of my ILL requests for materials likely available online, were returned unfilled than is typical
___Strongly disagree
___Disagree
___Neutral
___Agree
___Strongly agree

8. Did the library purchase digital access to any requested materials that you could not obtain through ILL or your regular channels? Check all that apply.
___Purchased directly from publisher
___Requested material through an existing relationship with a third-party content provider (e.g. ReprintsDesk)
___Contracted with a new content provider
___We didn't purchase any digital access/materials
___Other__________

9. Did you have access to your print collection from March to August 2020?
___Entire time
___Part of the time
___None of the time
___Not applicable; we don't have a print collection
___Other, please specify:

10. Were you filling ILL requests from your physical collection from March to August 2020?
___Entire time
___Part of the time
___None of the time
___Not applicable; we don't have a print collection

11. How did you provide ILL fills from your physical collection during that time? Check all that apply.
___Scanned selections of physical items for digital delivery
___Local courier
___Mail/commercial delivery
___Other __________
___Not applicable; we don’t have a print collection

12, Were you providing document delivery to your own patrons from March to August 2020?
___None of the time, neither print nor electronic
___Some of the time electronic, some of the time print
___All of the time electronic, some of the time print
___All of the time print, some of the time electronic
___All of time, both print and electronic

13. Were you providing physical items from your own or other libraries to your own patrons from March to August 2020?
___Entire time
___Part of the time
___None of the time
___Not applicable; we don’t have a print collection

14. If you did provide physical items to your own patrons, how did you do so? Check all that apply.
___Campus mail
___Curbside pick-up
___Expansion of existing mail-to-home (distance patron) service
___Launched new mail-to-home service
___In-library pickup
___We did not provide physical items
___Other __________

15. Were you accepting returns from March to August 2020?
___Entire time
___Part of the time
___We did not accept nor process any returns
___We did not want returns during that time, but items got returned anyway
___Not applicable; we do/did not loan physical materials.
___Other, please explain: ____

16, From March to August 2020, how did your library handle scanning of physical materials for ILL/DD?
___No change from pre-pandemic workflow
___Staff from other library departments were cross-trained
___Regular ILL staff on altered schedule
___Not applicable; we don’t provide scanning services
___Not applicable; we don’t have a print collection
___Other __________

17.How did your library handle reshelving of materials pulled for scanning between March-August?
___No change from pre-pandemic workflow
___ Delayed/backlog; did not develop a system to know what had already been pulled.
___Delayed/backlog; developed a system to know what had already been pulled. Please describe:
____
___Did not reshelve at all during this time; did not develop a system to know what had already been pulled.
___ Did not reshelve at all during this time; developed a system to know what had already been pulled. Please describe: ________________________________
___Not applicable

18. Has usage of your print collection for ILL/DD during the pandemic sparked evaluation of any of the following? Check all that apply.
___Gaps in your collection
___Titles/coverage unique to your collection
___Use of electronic titles
___Accessibility of print collection
___Organization of print collection
___Pursuit of new partnerships/cooperative agreements
___Staff cross-training
___ILL/DD processes
___Not applicable; we don't have a print collection
___Other: ____________

19. Did your interpretation of copyright guidance or practices related to CONTU guidelines change during the pandemic?
___No
___Yes - we have reinterpreted copyright guidance due to the extenuating circumstances of the pandemic
___Still under consideration
___Other, please specify ____

20. Did you join any new resource sharing groups, pods, consortia, or reciprocal agreements or invoke any emergency/buddy library agreements to gain additional access to ILL since March 2020? Check all that apply.
___No
___We joined new groups or signed new reciprocal agreements specifically to gain additional access during the pandemic
___We joined new groups or signed new reciprocal agreements during this time, but not specifically to gain additional access during the pandemic
___We invoked an existing emergency/buddy library agreement
___Other, please specify:

21. Has your policy regarding ILL charges for borrowing by your own library users changed since March 2020?
___No; we didn't change our policy of not charging

___No; we didn’t change our policy of charging
___Yes; we began providing ILL free of charge to all users
___Yes; we began providing ILL free of charge to some, but not all, users (e.g. free for students)
___Yes; we decreased our charges, but not to the point of being free
___Yes; we began charging
___Yes; we increased our charges from what we previously charged

22. Has your policy regarding charges for document delivery (DD) changed since March 2020?
___No; we didn’t change our policy of not charging
___No; we didn’t change our policy of charging
___Yes; we began providing DD free of charge to all users
___Yes; we began providing DD free of charge to some, but not all, users (e.g. free for students)
___Yes; we decreased our charges, but not to the point of being free
___Yes; we began charging
___Yes; we increased our charges from what we previously charged

23. Please share your sense of the financial impacts of ILL lending from March to August 2020.
___ILL revenue DECREASED substantially
___There was no major change in ILL revenue
___ILL revenue INCREASED substantially
___I do not have the information to answer this question.

24. Please share your sense of the financial impacts of ILL borrowing from March to August 2020.
___ILL expenditures DECREASED substantially
___There was no major change in ILL expenditures
___ILL expenditures INCREASED substantially
___I do not have the information to answer this question.

25. How were ILL staff involved in making decisions about any changes that happened with services?
26. Is there anything else you would like us to know or think we should have asked?
