# Supplementary material for: Interlibrary loan and document delivery in North American health sciences libraries during the early months of the COVID-19 pandemic
Source: J Med Libr Assoc. 2022 Jul 1;110(3):348–57. doi: 10.5195/jmla.2022.1452 (PMC9782377; doi:10.5195/jmla.2022.1452)
Supplement: Supplementary file 3 — Appendix C: List of Further Research Questions Raised by Respondents [file jmla-110-3-348-s03.pdf]

Appendix C: List of further research questions raised by respondents

Many respondents gave input for survey question 26 "Is there anything else you would like us to know or think we should have asked?" We grouped these suggestions into fifteen categories. In addition to responding to some of these as survey limitations in this article, we referred them to the two other research teams for their consideration.

Type of input / category	How addressed	Example of comment [Respondent]
Clarification needed / demarcation between ILL and DD	Limitation; improve primary data study forthcoming	In our hospital library, ILL is typically only print materials, while DD covers everything else -- this has nothing to do with whether the request is coming from an internal or external client. "
Response options / additional choices about increased services or audiences	Covered by other research surveys being done by others	….additional responses in Question 2, such as "increased services" and "new library users." In our case we garnered a whole new population of library users because [UNIT REDACTED] relocated their home-base to inside the Medical Library when their offices on the floors were displaced to become a closed COVID-19 unit.
Response options / distinguish print/physical journals from books	Limitation; improve primary data study forthcoming	Should have differentiated between print books and print journals in most questions - we have no print journals but still have a good-sized print book collection.
Question / when staff returned on-site	Not addressed; primary data study may ask this	When staff returned to on-site service
Question / size of staff, library or institution for comparison	Limitation; primary data study may ask this	Yes--library size, bed count, or other service-size indicator.
Question or Analysis / turnaround time pre-pandemic comparison.	Not addressed; primary data study may ask this	Overall, did you receive ILL or document delivery of borrowed requests in a timely fashion? Rationale: We found that many libraries were closed down completely, or had once-a-week access to their print collections, yet indicated that they had full access to their collections. Many others were auto-deflecting document requests, even when they HAD remote access to their electronic collections. It was very frustrating, as one never knew who/when would fill a request - even those that had a 4-day turnaround set. "<br>OR<br>has there been a change in timeliness of receiving ILLs

Question / fill volume and rates [This was the most requested question with eight offerings]	Secondary data study and primary data study will address	What was the percentage of ILL requests for items only in the print collection not filled? Or something about not having access to the Print collection to fill Docline requests
Question / perception of library services	Qualitative analysis theme	Has executive leadership become more aware of the services and skills you offer to the associates during the pandemic…?<br><br>Was there a sense of not fulfilling the Library mission, or of shortchan(g)ing patrons?
Question / staffing Issues	Qualitative analysis theme	I would like to know more about staff morale. How was it determined who would work remotely or work from onsite?
Question / workflow	Qualitative analysis theme	specific quarantining measures taken (if applicable), increased communication barriers (some institutes had no access to phone and were unresponsive via email)
Question / change in electronic usage	Not addressed; our focus was on materials not owned/licensed	Did you see an increase in the use of electronic materials during this time?
Question / impacts	Qualitative analysis theme	Were your library users negatively impacted by institutional changes due to the pandemic?
Question / user communications	Coded in the qualitative analysis but did not rise to the level of a theme.	How will charging our faculty/staff again be handled?
Question / changes over the course of the pandemic	Not addressed; single point of time survey	Did you resubmit previously rejected requests once lockdown measures loosened?
Question / ask opinion	Not addressed, though many used this question to share their opinions	An opinion question would have been nice.
