# Supplementary material for: Interlibrary loan and document delivery in North American health sciences libraries during the early months of the COVID-19 pandemic
Source: J Med Libr Assoc. 2022 Jul 1;110(3):348–57. doi: 10.5195/jmla.2022.1452 (PMC9782377; doi:10.5195/jmla.2022.1452)
Supplement: Supplementary file 4 — Appendix D: List of All Codes Considered and Applied to Data from Q25 and Q26 [file jmla-110-3-348-s04.pdf]

Appendix D: List of all codes considered and applied to data from Q25 and Q26

The codes that became themes or rolled up into a theme are marked [Theme]

Codes	Example
Access limitations to own library's physical collection [Theme]	Our main concern was not being able to fill from our print collection. However, staff was eventually able to begin coming sporadically to provide print resources. [Q26-R137]
Change in participation, demand, fill rate, turnaround times [Theme]	Since my library remained fully operational throughout the pandemic, the number of ILL Lend requests increased by approximately 50% compared to the previous year as other libraries were closed and requests got rerouted to me on DOCLINE. [Q26-R390]
Communication among libraries [Theme]	It's important to note the importance of the [LISTNAME 3 REDACTED] listserv- this was critical for us on a few occasions where patient care was involved. The timeliness of the responses from librarians on that listserv can not be overlooked, given the varied time zones and locations involved. [Q26-R39]
Consortial agreements	...early in pandemic weekly discussions with directors of libraries in our consortium were held to understand what was happening in our libraries regarding access to library, access to collections, etc. [Q25-R78]
DOCLINE customization, performance, status changes [Theme]	We are still happy with DOCLINE and do not want it to go away because it helps us participate in resource sharing with all libraries, not just academic, that have health science/biomedical collections. [Q26-R140]
Financial implications [Theme]	We made a pre-pandemic agreement with a third-party content provider and moved primarily to that document delivery model in March, 2020. [Q26-R184]
ILL staffing change and cross training [Theme]	Our Library was able to fill requests even though the Library was closed because of the ILL staff willingness to enter the building. Our ILL staff went the extra mile to fill requests. [Q26-R331]
License restrictions	we were not able to filled [sic] requests while working remotely from electronic resources with licenses that require "print then scan" [Q26-R211]
Negative impact of lack of access via ILL [Theme]	Our residents were able to visit nearby academic libraries but not any more on this difficult time. It is challenging for us and for anyone to locate needed items. [Q26-R382]
Perceived value of library services [Theme]	...What is extremely sad and dismaying and questionable, however, is that it appears many hospital libraries closed permanently during the pandemic, or after it was feasible to reopen--and this was at a time when they were, and are, needed more than ever before. [Q26-408]

Positive recognition	…The medical library was referred to as one of the gold stars of the Hospital during the pandemic. [Q25-R7]
Sharing system customization and performance, non-DOCLINE [Theme]	We participated in RapidILL's initiative to provide ILLs to libraries not in Rapid's system. We were happy to be able to provide this assistance. [Q26-R141]
Setting up/changing ILL technology for remote work [Theme]	We were told we had to work from home. Library access was left to my decision. I locked it except for badge access, ILL was totally limited to online resources. [Q25-R357]
User needs, attitudes, responses	...My "clients" understand the restriction for ILL and have been understanding. [Q26- R431]
Value of print collection [rolled up into "Perceived value of library services" theme]	This situation has helped us look at the print collection from a new angle as we are preparing for a new library space and needing to decide on how much print to take with us. [Q26-R428]
